# Water Adsorption on Hydrophilic Fibers and Porous and Deliquescent Materials: Cellulose, Polysaccharide, Silica, Inorganic Salt, Sugar Alcohol, and Amino Acid

**DOI:** 10.1021/acsomega.3c06642

**Published:** 2023-11-09

**Authors:** Masato Miyauchi

**Affiliations:** Tobacco Science Research Center, R&D Group, Japan Tobacco Inc., 6-2 Umegaoka, Aoba-ku, Yokohama, Kanagawa 227-8512, Japan

## Abstract

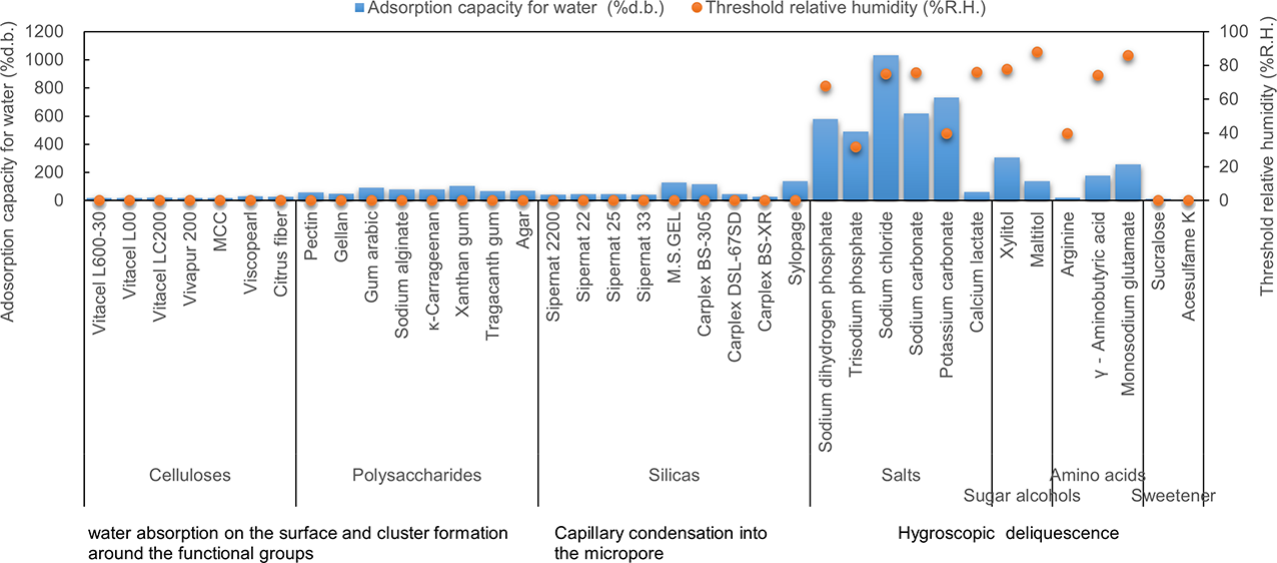

Water adsorption isotherms are systematically summarized by using celluloses and polysaccharides as hydrophilic crystal/amorphous materials with functional groups, silicas as hydrophilic porous materials, and inorganic salts, sugar alcohols, and amino acids as hygroscopic deliquescent materials. For hydrophilic fibers such as celluloses and polysaccharides, water was adsorbed on amorphous solids, and water clusters were formed around functional groups. For porous materials such as silicas, capillary condensation occurred in the micropores of silicas. For deliquescent materials such as inorganic salts, sugar alcohols, and amino acids, water adsorption rapidly increased stepwise at a specific threshold relative humidity, accompanied with a structure transformation to a liquid state. In addition, the water activity (Aw) of materials used in packed products was able to be estimated from the water adsorption isotherms of the pure component. This indicated that the deliquescent materials have a great effect on the depression of Aw for the suppression of microbial growth at an extremely high water content. The deliquescent materials could be useful to develop new environmentally and sustainable products and technologies with the mediation of water vapor and/or hydration.

## 1 Introduction

In recent years, hydrophilic materials have been gaining considerable interest for various water-related technologies in the fields of food, water, energy, biotechnology, environment, and medicine industries.^1,2^

First, product quality depends on the water content of materials used in packed products. Water vapor is then adsorbed on each component used in packed products and transfers outside the products through the packaging material based on the water adsorption isotherms and permeability through the packaging material.^3−5^ Undesirable migrations of water have caused serious deterioration in the quality. Water adsorption includes the adsorption on solid surfaces or into amorphous solids, the capillary condensation into micropores, the deliquescence, and the crystal hydration.^6^ The relationship between the water adsorption behavior and material structural properties for practical/commercial applications has been summarized.^2^ That is, the hygroscopic behavior is characterized by the profile of a water adsorption isotherm. Different types of materials exhibit different water capacities and shapes of water sorption isotherms. Therefore, systematic water adsorption isotherms of materials used in products are crucial to predicting the water content and quality of products during storage, transport, and marketing.

Second, the hygroscopic characteristics of materials used in packed products play an important role in the product preservation and hygiene of products during storage, transport, and marketing.^7,8^ Moist foods are liable to be contaminated by certain microorganisms, and their microbial growth requires nutrient and temperature to a certain extent as well as free water available to bacteria. Hence, FDA sets the water activity (Aw) of foods to 0.85 or less as a criterion for preventing food spoilage and microbial growth.^9^ In addition, salt preservation and sugar preservation methods are well-known to depress water activity.^7,8^ Herein, water activity is a water vapor pressure (*p*) in equilibrium with materials having a given water content divided by a saturated vapor pressure (*p*^o^) of water at the same temperature^10,11^
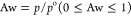


The water activity at a given water content can be determined by adsorption thermodynamics or solution thermodynamics.^11^ Therefore, to prevent microbial contamination and store products for a long time, the water activity should be set to 0.85 or less by adding inorganic salts and sugars.

Third, scientists have been interested in deliquescent materials. Studies on the hygroscopic behavior of deliquescent atmospheric aerosol particles such as NaCl have shown that an adsorbed water layer in core–shell form on the surface of airborne atmospheric aerosols lead to impacts on global climate change.^12,13^ Furthermore, applying deliquescence to products from a different point of view, a formulation has been filed in which an active ingredient is retained in a glassy transparent film formed by a deliquescent sugar alcohol and is gradually released in a sustained manner upon use as a product for transmucosal oral administration of the active ingredient.^14^ Qualitative and quantitative information regarding deliquescence may also be helpful for improving product qualities and adding a new feature with an associated self-organizing surface layer.

The objectives of this study are to summarize the water absorption–desorption isotherms for various hydrophilic materials such as celluloses and polysaccharides as hydrophilic crystal/amorphous materials with functional groups, silicas as hydrophilic porous materials, and inorganic salts, sugar alcohols, and amino acids as hygroscopic deliquescent materials, to discuss the hygroscopic characteristics of water adsorption behavior, and to provide useful information on the aspects of water-related utilizations with the mediation of water vapor and/or hydration.

## 2 Experimental Section

### 2.1 Materials

For celluloses, Vitacel powdered cellulose samples of L600-30 with a particle size of 30 μm, L00 with a particle size of 120 μm, and LC200 with a particle size of 300 μm and a Vivapur microcrystalline cellulose sample of 200 with a particle size of 190 μm were supplied by J. Rettenmaier (USA). Viscopearl porous cellulose was supplied by Rengo Co., Ltd. (Japan). Spherical microcrystalline cellulose (MCC) was supplied by Asahi Kasei Corporation (Japan).

For polysaccharides, pectin was purchased from Herbstreith & Fox GmbH & Co. KG Pektin-Fabriken, Neuenbürg (Germany) and gellan was purchased from San-Ei Gen F.F.I., Inc. (Japan). Gum arabic, sodium alginate, κ-carrageenan, xanthan gum, tragacanth gum, and agar were purchased from FUJIFILM Wako Pure Chemical Corporation (Japan). Citrus fiber was supplied by CP Kelco (USA).

For silicas, Sylopage was supplied by Fuji Silysia Chemical Ltd. (Japan), Sipernat 2200, Sipernat 22, Sipernat 25, and Sipernat 33 were supplied by Evonik (Germany), and Carplex BS-305, Carplex BS-XR, and Carplex DSL-67SD were supplied by Evonik (Japan). M.S.GEL was supplied by AGC Si-Tech Co., Ltd. (Japan).

For salts, sodium dihydrogen phosphate, potassium carbonate and sodium carbonate, and sodium chloride were supplied by Univar (UK) and trisodium phosphate was supplied by Israel Chemical Limited (Israel). Calcium lactate was supplied by Taihei Chemical Industrial Co., Ltd. (Japan).

For sugar alcohols, xylitol was supplied by Roquette (France) and maltitol was supplied by Caldic Nordics (Sweden).

For amino acids, arginine was purchased from Kyowa Hakko Bio. Co., Ltd. (Japan), γ-aminobutyric acid was purchased from Altaquimica (Spain), and monosodium glutamate was purchased from Marugo Corporation (Japan).

For sweeteners, acesulfame K was supplied by Univar and sucralose was purchased from FUJIFILM Wako Pure Chemical Corporation (Japan).

### 2.2 Water Adsorption Studies

Water vapor adsorption isotherms (at 25 °C) of samples were measured volumetrically by using an adsorption apparatus (Belsorp, MicrotracBEL Corp., Osaka, Japan). To remove any organic residues and moisture from the sample surface without surface reactions, the silica and others were evacuated below 0.1 Pa at 250 °C for 2 h and 105 °C for 1 h before adsorption, respectively. The preliminary tests confirmed that these desorption conditions were adequate to ensure that the adsorbed water could be measured with good reproducibility. The tolerance ranging from 0 to 90% R.H. *p*/*p*^o^ is within 0.3% of the reading variation per 300 s, and that ranging from 90% R.H. to ca. 95% R.H. is within 0.3 Pa of the reading variation per 300 s.

## 3 Results and Discussion

**Figure 1:**
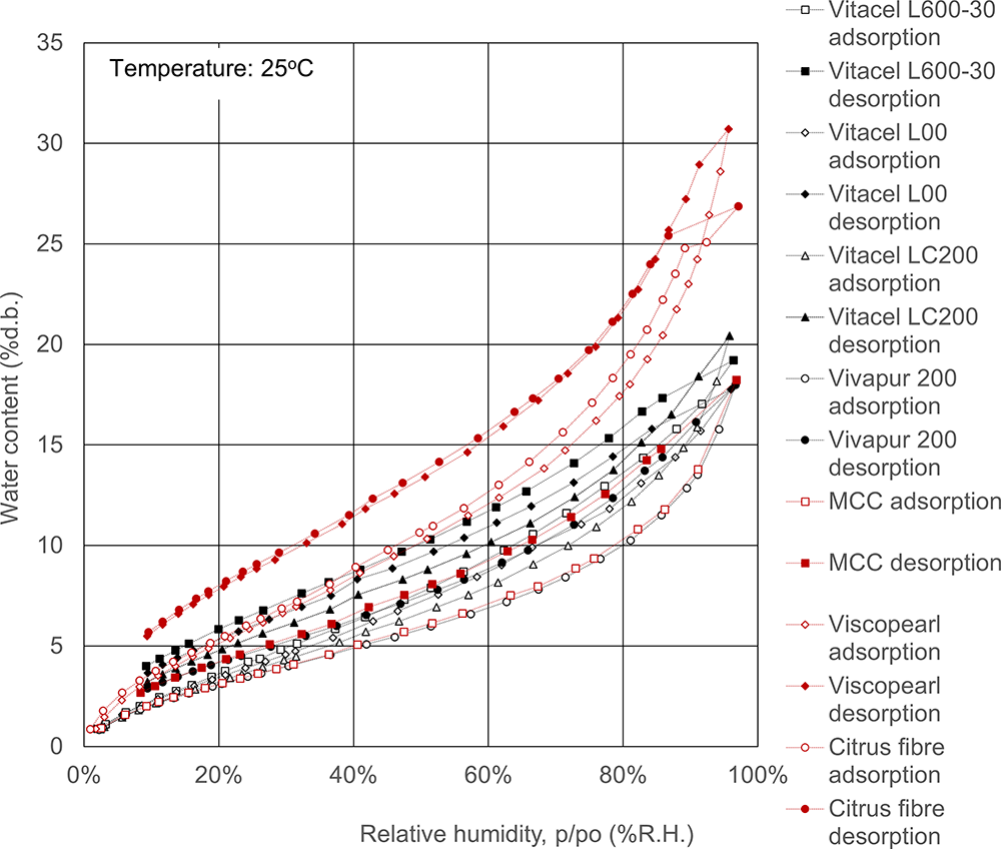
Water adsorption isotherms for celluloses. Open symbols, adsorption route; solid symbols, desorption route.

**Figure 2:**
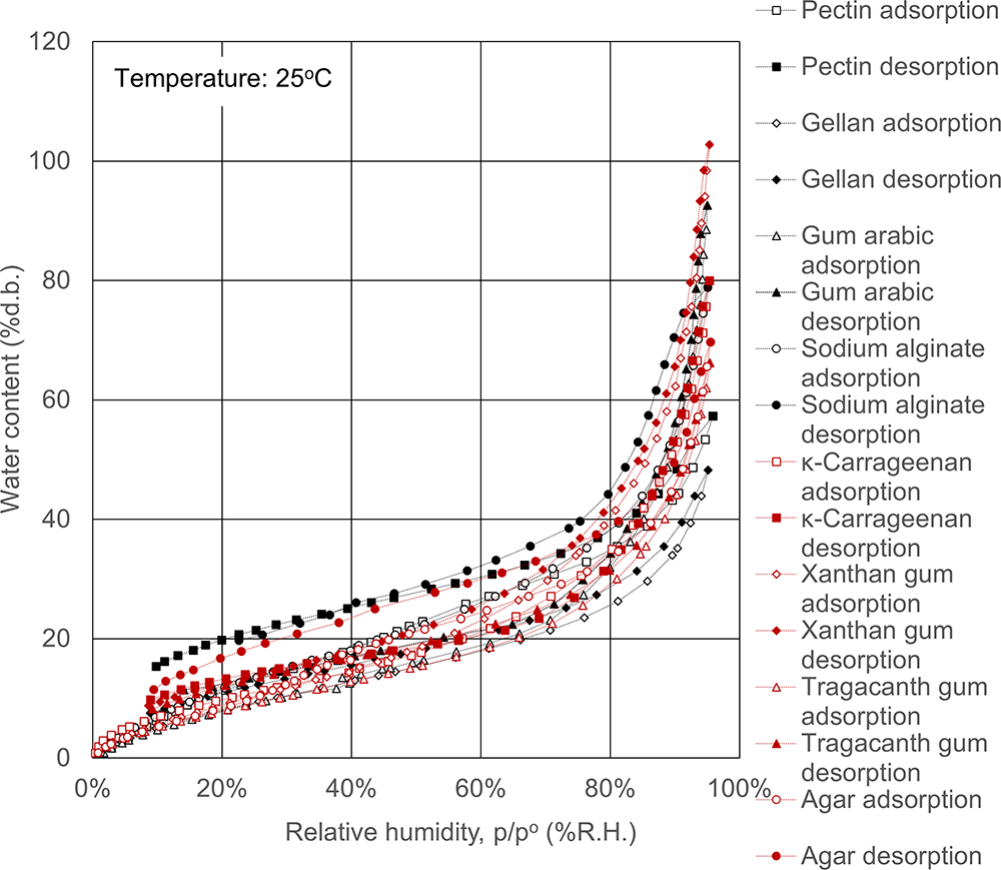
Water adsorption isotherms for polysaccharides. Open symbols, adsorption route; solid symbols, desorption route.

**Figure 3:**
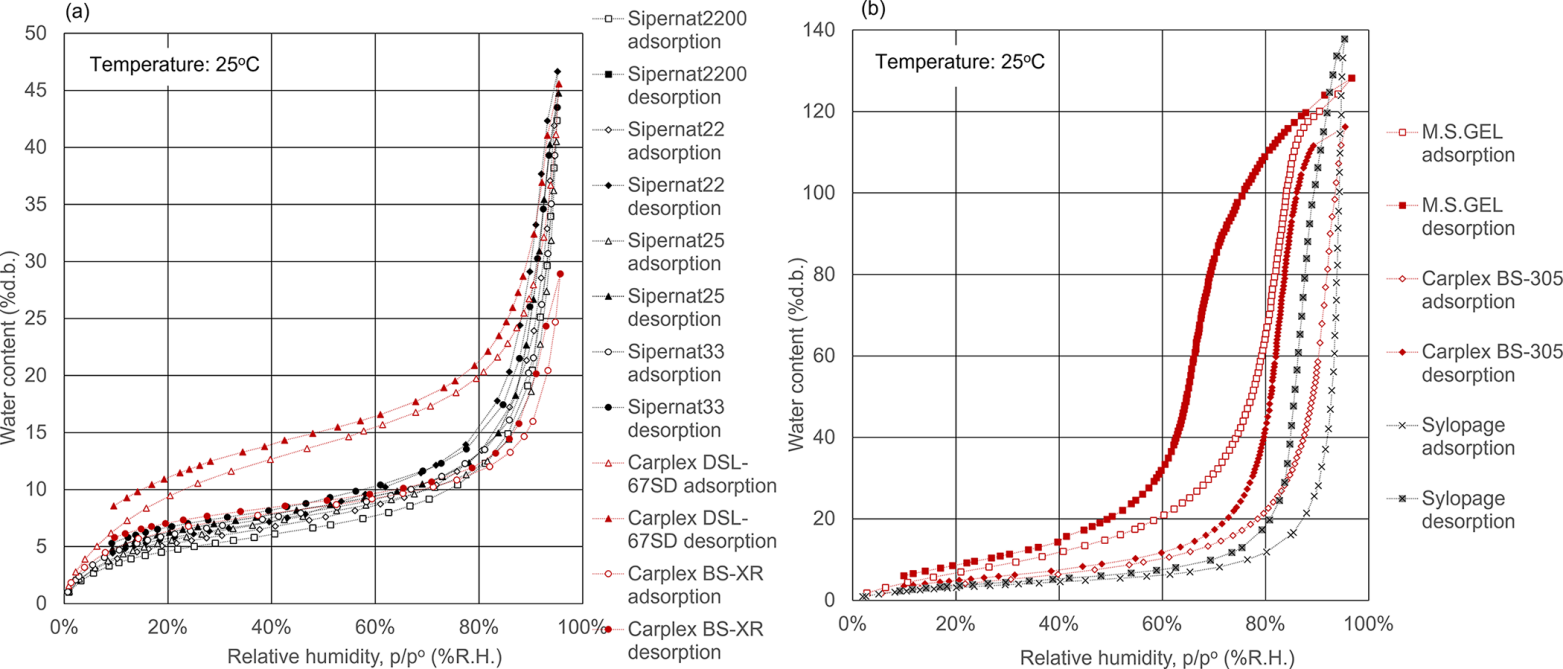
Water adsorption isotherms for (a) precipitated silicas and (b) gel-type silicas such as silica gel. Open symbols, adsorption route; solid symbols, desorption route.

**Figure 4:**
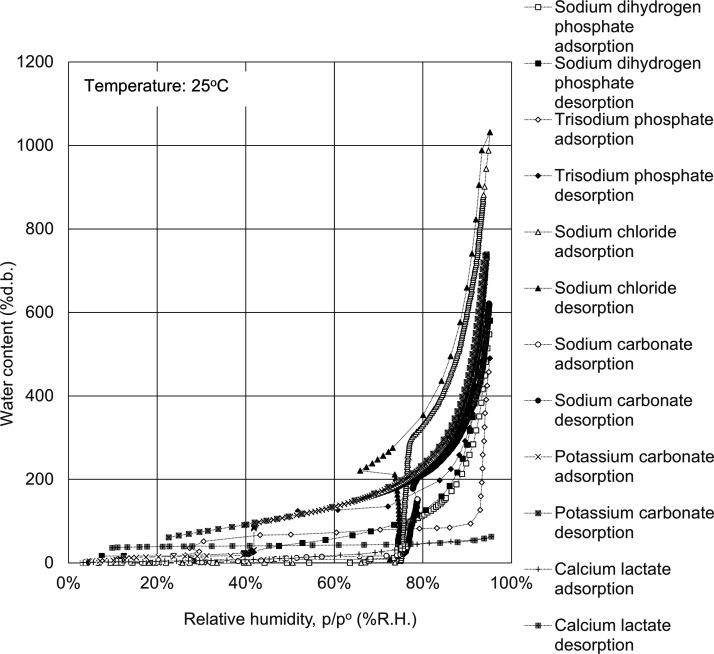
Water adsorption isotherms for salts. Open symbols, adsorption route; solid symbols, desorption route.

**Figure 5:**
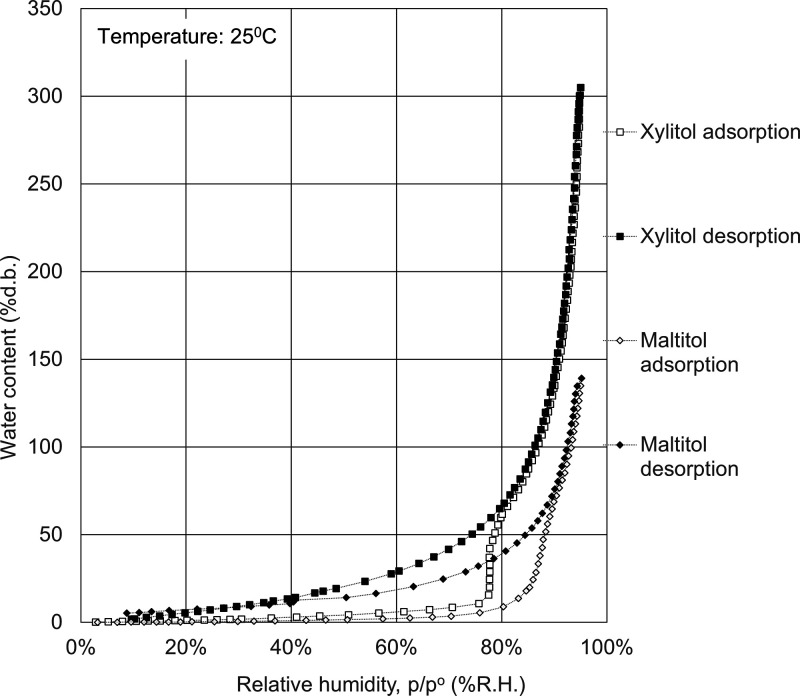
Water adsorption isotherms for sugar alcohols. Open symbols, adsorption route; solid symbols, desorption route.

**Figure 6:**
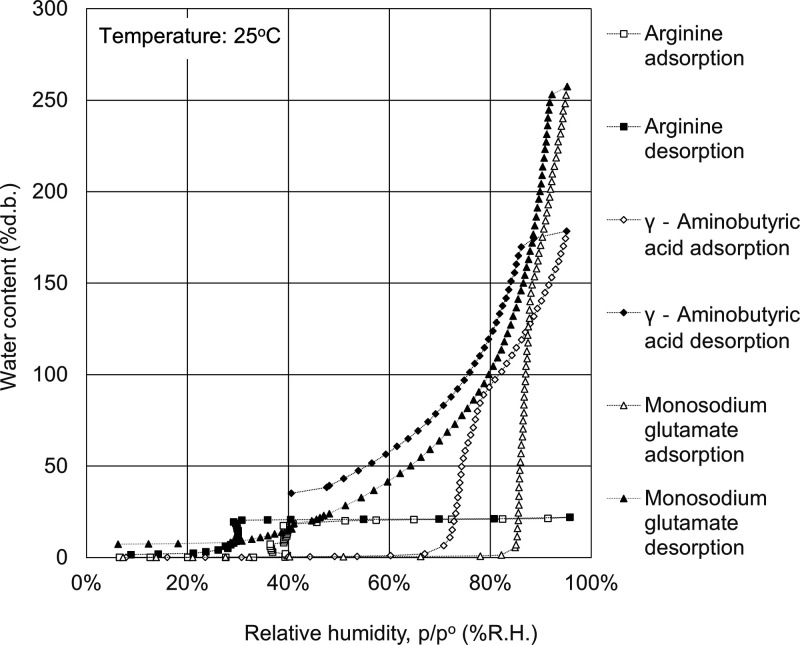
Water adsorption isotherms for amino acids. Open symbols, adsorption route; solid symbols, desorption route.

**Figure 7:**
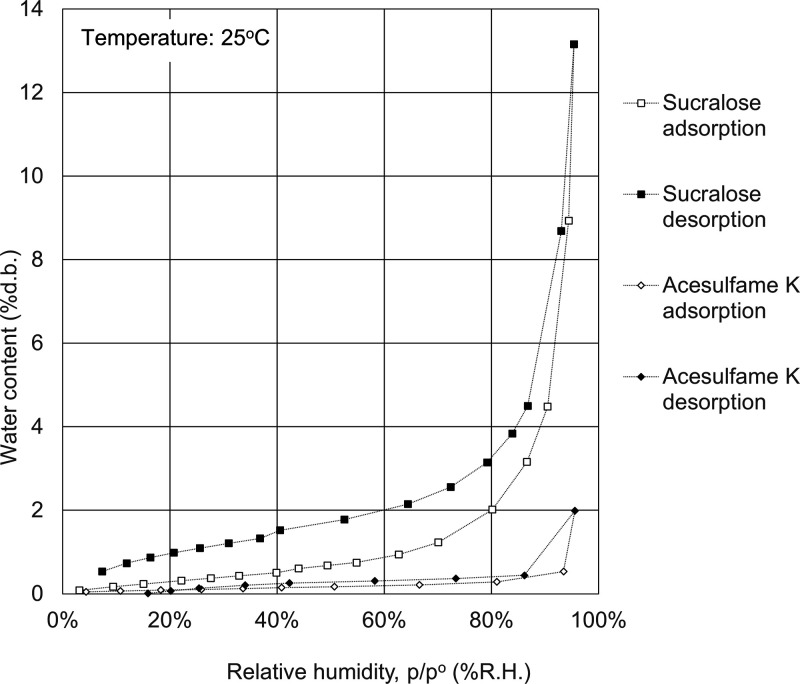
Water adsorption isotherms for sweeteners. Open symbols, adsorption route; solid symbols, desorption route.

The water adsorption isotherms of celluloses, polysaccharides, silicas, salts, sugar alcohols, amino acids, and others are shown in Figures 1–7.

### 3.1 Cellulose

**Figure 8:**
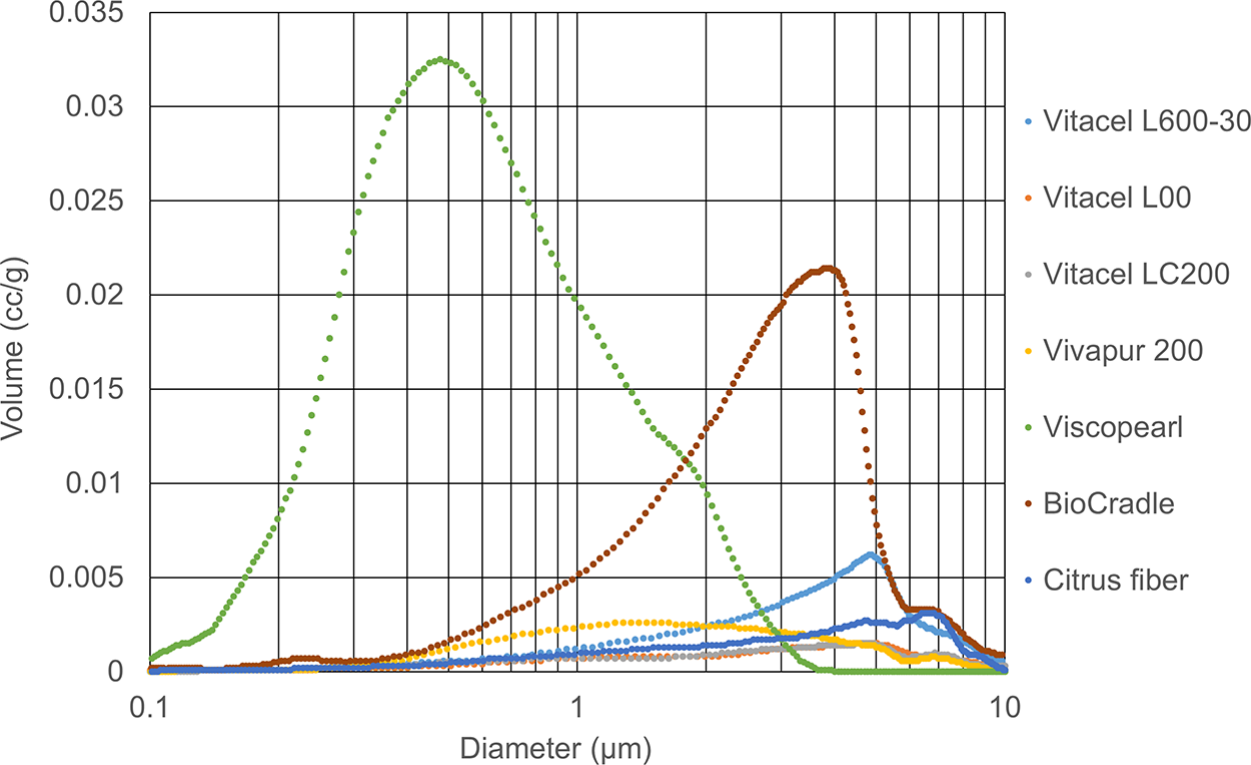
Macropore size distribution of celluloses obtained by mercury intrusion porosimetry.

As shown in the water adsorption isotherms of celluloses in Figure 1, they are increased in the area of zero relative humidity and the surface is hydrophilic.^3^ The water adsorption isotherms are nonlinear, typically sigmoidal in shape, and have been classified as type II.^15−17^ However, the water content of celluloses was less than those of others and was not related to the fiber length. It is reported that the water adsorption of cellulose is related to the cellulose crystallinity and pore volume.^18−20^ Although Tammelin et al.^21^ reported that a more crystalline cellulose film possessed nanoporosity, more surface area (more binding sites for water), and higher adsorption capacity of water when compared to an amorphous cellulose film, the lower water content of Vivapur and MCC with higher crystallinity^22^ has proven that they have less porosity, as shown in Figure 1. As shown in the macropore size distribution (i.e., greater than 50 nm) obtained by mercury intrusion porosimetry in Figure 8, Viscopearl, which is composed of regenerated celluloses made from viscose, is a porous powder with an average macropore volume equal to or greater than 8 times that of the other celluloses. However, the water content of Viscopearl is about 1.5 times more than that of the other celluloses and is less dependent on the macropore volume. In general, smaller molecules are more easily adsorbed into smaller pore spaces and are more strongly adsorbed due to micropore filling.^23,24^ This means that water capacity depends on the small pore volume ranging from micropores (i.e., less than 2 nm) to mesopores (i.e., 2–50 nm) along with the larger surface area.

Moreover, water adsorption isotherms of citrus fiber are also shown in Figure 1, and its water content was higher than that of cellulose, indicating that polysaccharides have a higher adsorption capacity of water than celluloses, as will be discussed later.

The macropore size distribution of the samples was determined by mercury intrusion porosimetry using a Pore Master 60-GT (Quantachrome Instruments, Boynton Beach, FL, USA); it can cover pores ranging from 6.5 nm (at a usual high pressure of 33000 psia) to 10 μm (at 20 psia). The sample weight is 0.2 g. The cell size is 10φ × 30 mm, and its volume is 0.5 cc. The contact angle of mercury is 140°, and the surface tension of mercury is 480 dyn/cm. The samples were evacuated for 105 °C and 2 h before measurement.

### 3.2 Polysaccharide

The potential uses of natural polysaccharides have recently attracted interest in water purification, drug delivery, tissue engineering, agriculture, and antimicrobial and biomedical applications.^1^ The water adsorption isotherms of the polysaccharides are shown in Figure 2. The water adsorption isotherms are typically sigmoidal in shape, have been classified as type II, and are more than those of cellulose.^16,17,22^ Polysaccharides have plenty of functional groups, such as a carboxyl group and amino group. Water adsorbed on the functional groups in the first step, and a water cluster was formed around the first adsorbed water molecules in the second step.^25−27^ Thus, it can be seen that the water content of the polysaccharide was relatively high, and for example, at the condition of an Aw of 0.85, the water content of the polysaccharide is 2 to 5 times greater than that of celluloses. The water adsorption isotherms of polysaccharide increased in the following order: gellan < tragacanth gum < pectin, gum arabic, κ-carrageenan, agar < sodium alginate, xanthan gum.

### 3.3 Silica

**Figure 9:**
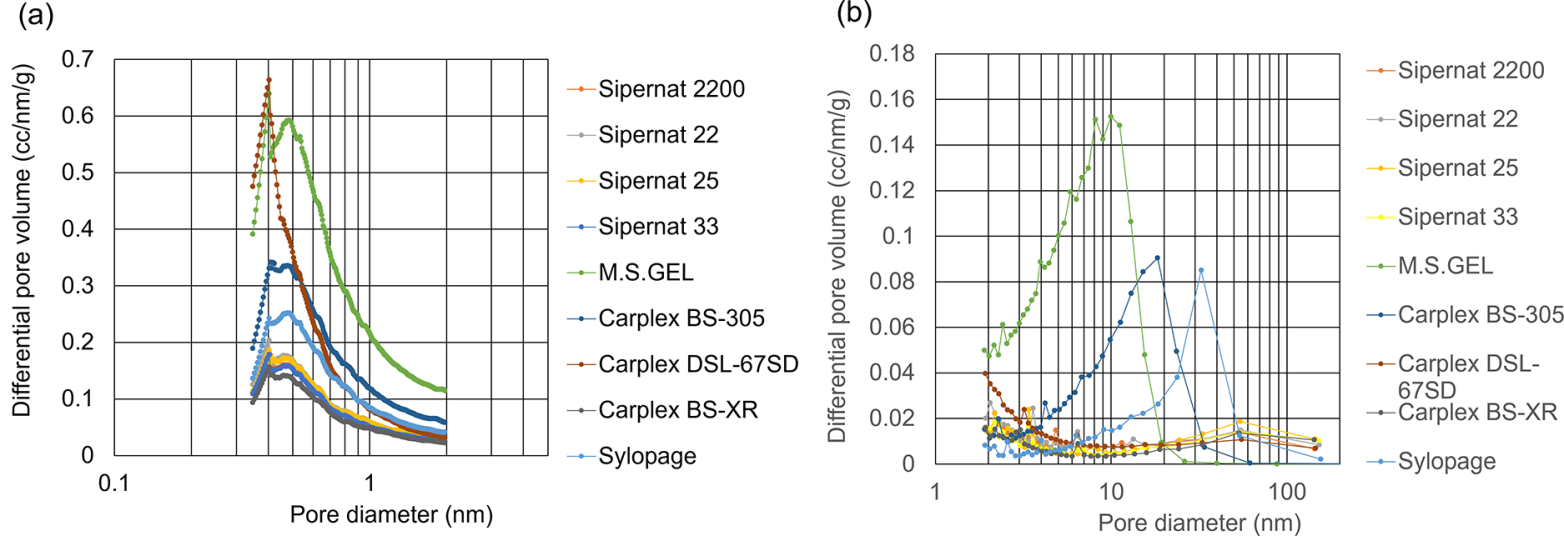
Micropore and mesopore pore size distribution of silica obtained using the (a) HK method and (b) BJH method.

Wet process silica has several types, such as precipitated and gel-type silica. The precipitated or gel-type silica was synthesized by reacting a sodium silicate solution with sulfuric acid under an alkaline or an acid condition, respectively. The precipitated silica is produced by neutralizing silica at reaction conditions of a relatively high temperature and an alkaline pH range. The primary particles grow at a faster rate and are highly condensed in flock-like form, which are then washed with water, filtered, and dried before consolidation. Alternatively, the gel-like silica is produced by consolidating the primary particles under the reaction conditions where the growth of the primary particles is suppressed by allowing the neutralization reaction to proceed in an acidic pH range, and the primary particles are transformed into a three-dimensional network structure. The porous gel is reacted using a mixing nozzle having a strong shear, and then, the gel-like silica is produced by washing with water, drying, and pulverizing.^28−30^ Recently, the precipitated silica having a structure close to that of gel-type silica has also been produced by using a synthesis method in which primary particles are grown in an aggregated state after causing aggregation while controlling the reaction temperature, pH, and salt concentration of the synthesis of silica primary particles and suppressing the growth of primary particles.^29,30^ The gel-type silica is featured by a high pore volume of micropores and mesopores without macropores (i.e., μm-sized voids).^30^ The pore properties and silanol group numbers of precipitated silica and gel-type silica are listed in Table 1, and the pore size distribution is shown in Figure 9. As shown in Table 1, M.S.GEL, Carplex BS-305, and Sylopage, which have a feature of gel-type silica, have plenty of micropores and mesopores without macropores, and these surface functional groups (i.e., silanol groups) are less than those of Sipernat, which has a feature of precipitated silica. In addition, as shown in Figure 3, the water adsorption isotherms of silicas exhibited type IV isotherms, and the water adsorption isotherms of M.S.GEL, Carplex BS-305, and Sylopage are higher than those of Sipernat at the same relative humidity. The equilibrium water contents of the gel-type silicas reached as much as 2–7 times as those of precipitated silica at a relative humidity of 85%. Thus, the high performance of water adsorption with the gel-type silica resulted from the capillary condensation into the abundance of micropores and mesopores with a hydrophilic surface. Also, since the hysteresis loop of the adsorption and desorption route was remarkably observed, it was proven to be difficult to desorb when water was adsorbed once.

**Table 1:** Specific Surface Area, Micropore, Mesopore, and Macropore Volume, and Number of Silanol Functional Groups

sample	*S*_BET_ (m^2^/g)	*V*_micro_ (cm^3^/g)	*V*_meso_ (cm^3^/g)	*V*_macro_ (cm^3^/g)	total pore volume (cm^3^/g)	silanol func. group (pcs/nm^2^)
Sipernat2200	224	0.11	0.78	0.99	1.79	1.5
Sipernat22	248	0.12	0.84	1.38	2.24	1.5
Sipernat25	242	0.11	0.95	1.62	2.59	1.5
Sipernat33	219	0.10	0.73	1.70	2.45	1.5
M.S.GEL	882	0.42	1.60	0.01	1.65	0.7
Carplex BS-305	474	0.23	1.52	0.02	1.55	0.6
Carplex DSL-67SD	502	0.23	0.70	1.04	1.87	1.0
Carplex BS-XR	196	0.09	0.65	1.59	2.27	1.8
Sylopage	348	0.17	1.91	0.37	2.30	not measured

The specific surface area (*S*_BET_) of the samples was determined from N_2_ adsorption–desorption isotherms obtained at 77 K using a BELSORP-max (BEL Japan, Osaka, Japan); the specific surface areas were calculated using the Brunauer–Emmett–Teller (BET) method. The pore volume and pore size distribution were determined using the Barrett–Joyner–Halenda (BJH) for mesopores and macropores and Horvath–Kawazoe (HK) methods for micropores.^40^ A micropore, mesopore, and macropore are defined as a pore with a diameter of ≤2 nm, 2–50 nm, and ≥50 nm, respectively. The total pore volume was assessed to be the N_2_ adsorption volume at a relative pressure (*p*/*p*^o^) of 0.99. The number of silanol groups (Sears number, ρ(pcs/nm^2^)) on the surface of silica by titration was determined by a method as developed by Sears.^41^ The Sears number was given as
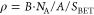
where *B* is the consumed volume of sodium hydroxide between pH 4 and pH 9 (mL), *N*_A_ is Avogadro’s number, *A* is the sample weight (g), and *S*_BET_ is the specific surface areas (nm^2^/g).

### 3.4 Inorganic Salt

The adsorption isotherms of salts are shown in Figure 4 and exhibited type IV isotherms. Water adsorbs significantly more on the inorganic salts than on the others in the range of over 80% R.H. The adsorption capacity of water can reach as high as 10 times the sample weight for sodium chloride, 7 times the amount for potassium carbonate, 6 times the amount for sodium carbonate and sodium dihydrogen phosphate, and 5 times the amount for trisodium phosphate. These findings show that the inorganic salts are hygroscopic deliquescent materials and have the highest adsorption capacity of water. The water adsorption isotherm gradually rises to a threshold relative humidity, rapidly increases stepwise when the relative humidity reaches a threshold level, and then rises gradually until it becomes saturated, while the water desorption isotherm decreases monotonically with decreasing relative humidity even when the relative humidity reaches the threshold level.^31^ As shown in Figure 4, the threshold relative humidity is 75% for sodium chloride, in agreement with the previous reports.^32,33^ Moreover, the threshold relative humidity is 76% for sodium carbonate, 40% for potassium carbonate, 68% for sodium dihydrogen phosphate, and 32% for trisodium phosphate. It is reported that a phase change of the inorganic salts occurs from solid to liquid by the absorption of water vapor at the threshold relative humidity.^34^ Additionally, it has been reported that in metal/metal oxides, silicon/silicon oxides, fluorides, and two-dimensional materials, the liquid water structure is superior to the solid-like structure, also denoted as the ice or ordered structure, over 30–60% R.H.^35^ and the solid network swelling would occur due to electrical repulsion between the functional groups or electrostatic interaction at high water contents.^11^ The findings indicated that at the threshold relative humidity, in the adsorption route, the structure swells and transformation to a liquid state occurs, but even at the threshold relative humidity in the desorption route, the inorganic salts do not transform to the initial solid state and keep the liquid state for a while, which is responsible for the adsorption hysteresis loop in the isotherms.

### 3.5 Sugar Alcohol

The water adsorption isotherms of sugar alcohols are shown in Figure 5. Although the water adsorption isotherms of xylitol are reported to be nonlinear, typically sigmoidal in shape, and have been classified as type II,^17^ the water adsorption isotherms of xylitol and maltitol exhibited combined isothermal adsorption isotherms (between type II and type IV) and rapidly increased stepwise at the threshold relative humidity, in a similar behavior to the salts, as shown in Figure 5. Sugar alcohols are also hygroscopic deliquescent materials. The adsorption capacity of water can reach as high as 3 times the sample weight for xylitol and 1.4 times the amount for maltitol. The threshold relative humidity is 77% for xylitol and 87% for maltitol. Sugar alcohols have a high adsorption capacity of water.

### 3.6 Amino Acid

To understand the deliquescence characteristics of amino acids at an extremely high relative humidity ranging from 80 to 99.5%, the water adsorption isotherms were measured even when liquefied, as will be mentioned later. The water adsorption isotherms of amino acids are shown in Figure 6. The hygroscopicity of amino acids including hysteresis depends on the interactions between polar groups such as coordination into the crystal structure.^36^ The hygroscopicity of amino acids in atmospheric aerosols was also reported to be influenced by whether or not crystallization and deliquescence occur.^37^

First, the water adsorption isotherms of γ-aminobutyric acid and monosodium glutamate exhibited the combined isothermal adsorption isotherms (between type II and type IV) and increased stepwise at the threshold relative humidity. The adsorption capacity of water can reach as high as 1.7 times the sample weight for γ-aminobutyric acid and 2.5 times the sample weight for monosodium glutamate. The threshold relative humidity is 74% for γ-aminobutyric acid and 86% for monosodium glutamate. γ-Aminobutyric acid and monosodium glutamate are deliquescents and have a high adsorption capacity of water. It is reported that a deliquescent material exhibits a solid to solution phase transformation when the relative humidity reaches its threshold, with kinetics increasing as the relative humidity further increases.^38^ The findings showed that their water uptake is attributed to liquefaction over the threshold relative humidity. Both γ-aminobutyric acid and monosodium glutamate changed from white powder to viscous transparent fluid when deliquescence occurs at a high relative humidity (see also Figure S1 in the SI). When evaluated using a spectrophotometer (CM-5, Konica Minolta, Japan), γ-aminobutyric acid and monosodium glutamate after the water adsorption experiments are shown as transparent. After a while, γ-aminobutyric acid remains a viscous transparent film, while monosodium glutamate becomes an opaque solid (see also Figure S1 in the SI). The stability of deliquescence appears to depend on the stability of a quasi-stable state of the liquid state.

Second, although the water adsorption isotherm of arginine increases stepwise at the threshold relative humidity of 40%, the adsorption capacity of water is just 0.2 times the sample weight and has a low adsorption capacity of water.

### 3.7 Artificial Sweetener

As shown in the water adsorption isotherms of sweeteners in Figure 7, water hardly adsorbed on the artificial sweeteners (sucralose and acesulfame K) and the water adsorption isotherms of sweeteners exhibited type II isotherms. Artificial sweeteners have low adsorption capacity of water.

### 3.8 Application to Water Activity Estimation of Products

For the suppression of microbial growth, a mass fraction of mixture with an Aw of 0.85 or less at a given water content is essential to be fixed experimentally by adjusting deliquescent materials such as salt, sugar, and the like. When the water activity can be calculated by using the water adsorption isotherm from various mass fractions, the mass fraction having the Aw of 0.85 or less can be efficiently determined.

Kumagai et al.^11^ reported the measurement of water sorption isotherms of superabsorbent polymers, and their water activity was evaluated by solution thermodynamics to prevent microbial contamination during transportation or storage. However, since it is difficult to apply solution thermodynamics to solids that do not dissolve in water, the water activity was determined by adsorption thermodynamics. In this study, the total water content of multicomponents at a given Aw is equal to a weighted average of water contents of pure components at a given Aw, as reported in a previous study.^39^ That is, the water activity of the mixture is calculated by summing the multiplication of each water content by its mass fraction in the pure component.

The estimation of Aw was proven as follows. First, a mass fraction of the mixture was determined in equilibrium with the Aw of 0.85 under the condition of a water content of 50% wet basis (w.b.) by experimentally repeating the mixing and the measurement of Aw with a LabMaster-aw NEO (Novasina, Lachen, Schweiz). Second, the water content of a pure component in equilibrium with the relative humidity of 85% was obtained from the water adsorption isotherm, and the total water mass of the mixture was calculated by summing the water mass in each component, as listed in Table 2. The estimated water content (% w.b.) becomes 51% w.b. and 54% w.b. in the adsorption and desorption routes, respectively, and almost agrees with the experimental value (50% w.b.).

**Table 2:** Estimated Water Content at a Water Activity of 0.85

materials	composition mass (g)	estimated water mass on the adsorption route (g)	estimated water mass on the desorption route (g)
Vitacel L600-30	56.6	8.44	9.78
sodium dihydrogen phospahte	18.4	28.29	33.92
potassium carbonate	15.2	44.31	45.93
sodium chloride	5.7	24.07	26.19
calcium lactate	1.5	0.75	0.74
acesulfame K	2.0	0.01	0.01
sucralose	0.2	0.01	0.01
gellan	0.4	0.12	0.15
total	100.0	106.00	116.74
water content (% w.b.)		51	54

Ghorab et al.^31^ reported that synergistic interactions, among the deliquescent components partially or completely dissolved, occur among the blending of NaCl and maltodextrins when the deliquescent components such as sodium chloride were in contact with or in close proximity to the amorphous maltodextrin particles. However, as the water content of the mixture can be calculated by the pure adsorption isotherm, the blending of the deliquescent solids and the others has little synergistic effect. This indicated that plenty of water adsorbs on the deliquescent solids around the Aw of 0.85, except for maltitol with a threshold relative humidity of 87% and sodium glutamate with a threshold relative humidity of 86%, and a further increase in the equilibrium water content by blending does not occur.

The water contents (% w.b.) in equilibrium with the Aw of 0.85 in the adsorption and desorption routes are listed in Table 3. By utilizing these values, it is possible to calculate the mass fraction of mixture depressing the Aw equal to or less than 0.85 to suppress microbial growth during transportation, storage, and marketing. This demonstrated that the deliquescent materials, i.e., the inorganic salts, sugar alcohols, and amino acids, can have the greatest effect on the depression of the Aw.

**Table 3:** Estimated Water Content at a Water Activity of 0.85

		water content (% d.b.)	
materials		adsorption route	desorption route
cellulose	Vitacel L600-30	15	17
	Vitacel L00	14	16
	Vitacel LC200	13	17
	Vivapur 200	11	14
	MCC	12	15
	Viscopearl	20	26
	citrus fiber	22	25
polysaccharide	pectin	38	44
	gellan	29	35
	gum arabic	40	48
	sodium alginate	44	57
	κ-carrageenan	42	44
	xanthan gum	49	52
	tragacanth gum	35	39
	agar	38	44
silicas	Sipernat 2200	15	16
	Sipernat 22	17	19
	Sipernat 25	14	16
	Sipernat 33	16	18
	M.S.GEL	107	117
	Carplex BS-305	32	92
	Carplex DSL-67SD	22	24
	Carplex BS-XP	13	14
	Sylopage	16	43
salts	sodium dihydrogen phosphate	153	184
	trisodium phosphate	84	225
	sodium chloride	421	458
	sodium carbonate	256	265
	potassium carbonate	291	301
	calcium lactate	50	49
sugar alcohols	xylitol	87	91
	maltitol	19	54
amino acids	arginine	21	21
	γ-aminobutyric acid	114	159
	monosodium glutamate	6	136
sweeteners	sucralose	3	4
	acesulfame K	0.4	0.4

## 4 Conclusions

The water adsorption–desorption isotherms of celluloses, polysaccharides, silicas, salts, sugar alcohols, amino acids, and others were measured. Celluloses and polysaccharides were used as the hydrophilic crystal or amorphous materials with functional groups, silicas were used as the hydrophilic porous materials, and the inorganic salts, sugar alcohols, and amino acids were used as the deliquescent materials.

For the celluloses and polysaccharides, polysaccharides with plenty of functional groups have higher adsorption capacity of water than celluloses, with associated cluster formation. For the silicas, the adsorption capacity of water depends on their pore volume based on capillary condensation into the micropore. The gel-like silicas that were synthesized under acidic conditions have a greater pore volume of micropores and mesopores without macropores than the precipitated silica that was synthesized under alkaline conditions. The equilibrium water contents of the gel-type silicas showed 2–7 times more than those of the precipitated silicas at a relative humidity of 85%. For the hygroscopic deliquescent materials, the inorganic salts induced the water content uptake above the threshold relative humidity, water is absorbed on them from 5 to 10 times their own mass, and the threshold relative humidity ranges from 32 to 75%, with associated structural transformation. Sugar alcohols and some amino acids also induced the water content uptake and have a water adsorption capacity several times their own mass, but their threshold relative humidity of over 77% is relatively high compared with the inorganic salts. Maltitol with a threshold relative humidity of 87% and monosodium glutamate with a threshold relative humidity of 86% have little effect on depressing the Aw below 0.85.

The water activity of products can be estimated using the water adsorption isotherms of pure material used in products and the effect on depressing the Aw for the suppression of microbial growth increased in the following order: celluloses < silicas < polysaccharides < sugar alcohols < amino acids < inorganic salts. In addition, γ-aminobutyric acid and monosodium glutamate can form a self-organizing surface layer on the product with the mediation of water vapor and/or hydration, which makes it possible to improve product qualities and add a new feature.

## Supporting Information Available

Appearance of amino acids immediately and preserved for 9 days after the water adsorption experiment (PDF)
