# Supplementary material for: Water Adsorption on Hydrophilic Fibers and Porous and Deliquescent Materials: Cellulose, Polysaccharide, Silica, Inorganic Salt, Sugar Alcohol, and Amino Acid
Source: ACS Omega. 2023 Nov 9;8(46):44212–20. doi: 10.1021/acsomega.3c06642 (PMC10666253; doi:10.1021/acsomega.3c06642)
Supplement: Supplementary file 1 — ao3c06642_si_001.pdf [file ao3c06642_si_001.pdf]

Supporting Information for

Water adsorption on hydrophilic fibers, porous and deliquescent materials: Cellulose, Polysaccharide, Silica, Inorganic salt, Sugar alcohol and Amino acid

Masato Miyauchi

Tobacco Science Research Center, R&D Group, Japan Tobacco Inc., 6-2 Umegaoka, Aoba-ku, Yokohama, Kanagawa, 227-8512, Japan.

* Corresponding Authors: Masato Miyauchi, E-mail: masato.miyauchi@jt.com Tel: +81-80-1290-4329

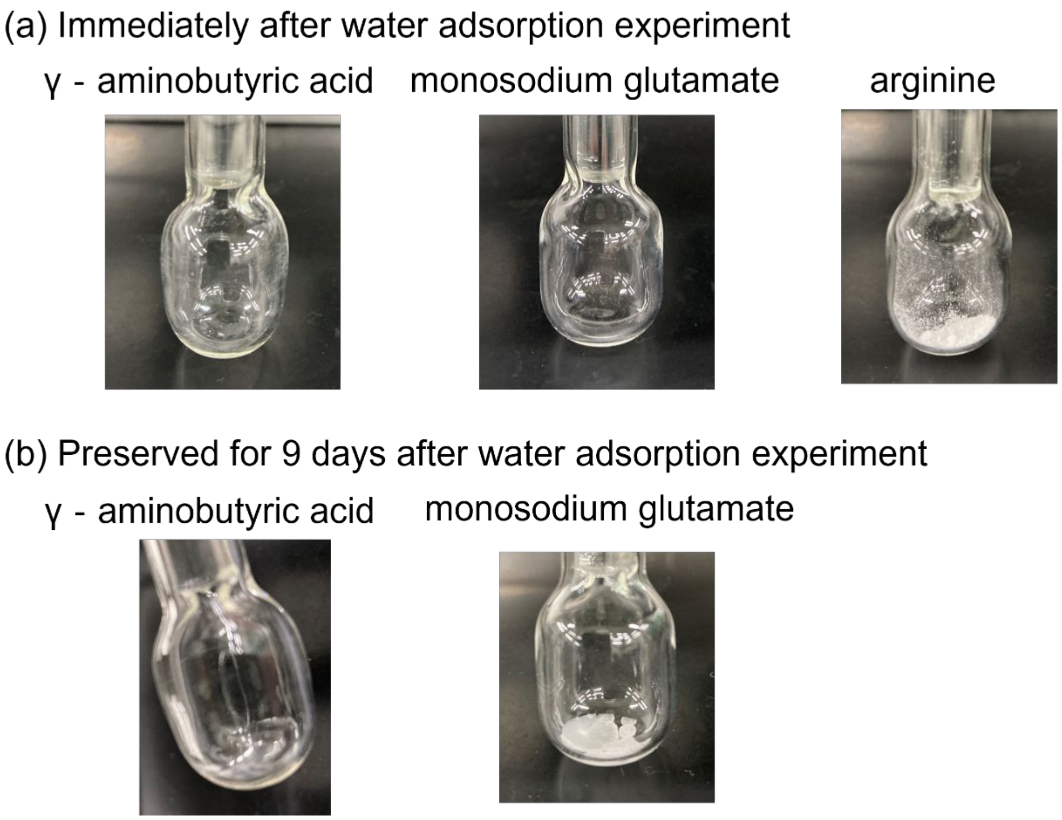

Fig.S1 Appearance of γ-aminobutyric acid, monosodium glutamate and arginine (a) immediately and (b) preserved for 9 days after water adsorption experiment.
